# Pathogenic Roles of *Fusobacterium nucleatum* in Colorectal Cancer: From Strain Heterogeneity to Host–Pathogen Interactions

**DOI:** 10.3390/pathogens15050483

**Published:** 2026-04-30

**Authors:** Ruihong Xiao, Yanrui Bai, Wenxiu Liu, Hui Sun

**Affiliations:** 1 Cuiying Biomedical Research Center, The Second Hospital & Clinical Medical School, Lanzhou University, Lanzhou 730030, China; xiaorh2024@lzu.edu.cn (R.X.); wxiuliu@163.com (W.L.); 2 High Altitude Medical Research Center, Xining 810000, China; baiyr20@lzu.edu.cn

**Keywords:** *Fusobacterium nucleatum*, colorectal cancer, pathogen–host interactions, microbial heterogeneity, Fna C2, tumor microenvironment, immune evasion, precision intervention

## Abstract

*Fusobacterium nucleatum* (Fn) has emerged as one of the most extensively studied tumor-associated opportunistic pathogens in colorectal cancer (CRC). The central question in Fn–CRC research has shifted from species-level detection or enrichment toward identifying specific lineages with enhanced persistence and tumor-promoting potential under defined host and ecological contexts. Accumulating evidence suggests substantial heterogeneity within Fn at the subspecies and clade levels. Among these, the *F. nucleatum* subsp. *animalis* C2 (Fna C2) lineage has been proposed as a candidate high-risk clade with potentially greater adaptability to the gastrointestinal tract and tumor microenvironment. However, current support for Fna C2 is derived mainly from ecological enrichment, comparative genomics, inferred metabolic features, and limited functional observations, while direct clinical and mechanistic validation at the clade level remains limited. Fn has been implicated in CRC progression through multiple interconnected processes, including adhesion and colonization, host signaling activation, inflammatory amplification, immune suppression, and metabolic adaptation. Notably, these pathogenic outputs are unlikely to be uniformly distributed across all Fn lineages, but instead appear to be shaped by the combined influence of bacterial lineage, host molecular context, microbial community structure, and spatial organization within the tumor microenvironment. In this review, we summarize the lineage heterogeneity of Fn, its association with CRC, and the underlying host–pathogen interaction mechanisms. We further discuss implications for high-resolution stratification, risk classification, and clinical translation, emphasizing the need to move from species-level associations toward lineage-resolved and context-aware frameworks.

## 1. Introduction

Colorectal cancer (CRC) remains one of the leading causes of cancer-related morbidity and mortality worldwide [1]. Its development and progression arise from complex interactions between host genetic susceptibility and multiple environmental factors, including dietary patterns, chronic inflammation, metabolic dysregulation, and alterations in the gut microbiota [2,3,4,5]. With the rapid development of high-throughput sequencing, multi-omics integration, and spatially resolved technologies, the gut microbiome is no longer viewed merely as a bystander in tumorigenesis, but is increasingly recognized as an important factor influencing CRC initiation, progression, and therapeutic response [5,6].

Among the microbial taxa associated with CRC, *Fusobacterium nucleatum* (Fn) is one of the most extensively investigated and consistently reported species. Fn is enriched in tumor tissues, has been associated with poor clinical outcomes, and exhibits tumor-promoting effects in both in vitro and in vivo models [7,8,9,10]. Accumulating evidence suggests that Fn should not be regarded solely as a commensal member of the oral microbiota, but rather as an opportunistic pathogen with the capacity to expand across ecological niches and participate in tumor-associated pathological processes [11]. In addition, Fn may disseminate along the oral–gut axis and contribute to tumor-related pathophysiology in extra-oral environments [10,12].

Despite substantial evidence linking Fn to CRC through tissue enrichment, clinicopathological associations, and experimental models, a key conceptual gap remains in the current field. Many previous studies have treated “Fn positivity” as a species-level signal, without adequately resolving the heterogeneity that exists among subspecies, clades, and individual strains [13,14,15]. As a result, potentially important differences in ecological adaptation, host interaction, and pathogenic potential may have been obscured. Accordingly, the key challenge in Fn–CRC research is no longer simply to determine whether Fn is present or enriched, but rather to identify which candidate high-risk lineages exhibit biologically and clinically relevant effects under specific host and ecological conditions [16]. In this context, the present review focuses on the heterogeneity of Fn and systematically summarizes its association with CRC as well as the underlying host–pathogen interaction mechanisms. We further discuss how host background and ecological context may shape Fn-associated pathogenic outputs, and consider the implications of these insights for risk stratification and clinical translation.

## 2. Biological Characteristics and Heterogeneity of *Fusobacterium nucleatum*

### 2.1. Taxonomy and General Biological Characteristics

*Fusobacterium nucleatum* (Fn) belongs to the phylum *Fusobacteriota*, class *Fusobacteriia*, order *Fusobacteriales*, family *Fusobacteriaceae*, and genus *Fusobacterium*, and is one of the most extensively studied and clinically relevant members of this genus. Fn is a Gram-negative, obligate anaerobic, non-spore-forming fusiform bacterium that primarily colonizes low-oxygen niches such as subgingival plaque and dental biofilms, where it plays an important organizational role in the oral microbial ecosystem [11,17].

Unlike many oral bacteria that are largely confined to local commensal states, Fn exhibits both symbiotic features and opportunistic pathogenic potential. Under homeostatic conditions, it can persist as a resident member of the oral microbiota. However, under conditions such as microbial dysbiosis, impairment of mucosal barrier integrity, or immune dysregulation, Fn is not necessarily restricted to its original oral niche, but may expand into other tissue environments and participate in pathological processes [10,11,18]. Accordingly, Fn may be more appropriately regarded as an opportunistic pathogen with the capacity for cross-niche expansion, rather than as a simple oral commensal.

### 2.2. Distribution in the Oral Cavity and Gut and Its Pathogenic Basis

Within the oral microbiota, Fn can bridge early and late colonizers and is therefore considered a key community-organizing species that contributes to the maturation and spatial architecture of multispecies biofilms [17]. Although Fn is not typically a stable dominant member of the gut microbiota, it may achieve persistent colonization in aberrant ecological settings under conditions such as dysbiosis, barrier disruption, and chronic inflammation, where it can be retained through its adhesive properties, participation in biofilm formation, and environmental adaptability [10].

Thus, the significance of Fn in the gut lies not merely in its detection, but in whether it can persist under specific pathological conditions and contribute to local disease processes. Importantly, this oral–gut distributional difference suggests that distinct Fn subgroups may not be equivalent in terms of environmental adaptability or pathogenic potential, thereby providing a basis for understanding its relationship with CRC at a higher level of resolution [13,16].

### 2.3. Subspecies and Clade-Level Heterogeneity

Emerging evidence suggests that Fn is not a genetically or functionally uniform species, but instead exhibits substantial heterogeneity at the subspecies and clade levels [14,15]. Different subgroups may vary in ecological adaptation, tissue distribution, metabolic characteristics, and potential pathogenicity. Therefore, the relationship between Fn and CRC may not be adequately explained by an average species-level effect, but may instead be driven by a limited number of higher-risk subgroups that are better adapted to the intestinal environment and possess stronger tumor-promoting potential [16,19]. The distribution of different Fn subspecies is not consistent across ecological niches and disease settings. For example, *F. nucleatum* subsp. *polymorphum* is more commonly detected in oral plaque, whereas *F. nucleatum* subsp. *animalis* appears to be more prevalent in odontogenic infections [20]. In CRC, although multiple subspecies can be detected, certain subgroups appear to show more pronounced enrichment and greater ecological adaptation [13,16]. These observations suggest that distinct Fn subgroups may differ in host adaptation and disease relevance.

Traditional subspecies classification captures part of the biological diversity within Fn, but it remains insufficient to explain the heterogeneous findings reported across studies and disease contexts. First, individual subspecies may still encompass clades with distinct ecological adaptability and pathogenic potential. Second, many previous studies have remained at the genus or species level, which may have led to biologically distinct subgroups being analyzed as a single entity [21]. Thus, the key to understanding Fn heterogeneity lies not simply in distinguishing subspecies names, but in identifying functionally relevant lineages associated with CRC at higher taxonomic resolution.

### 2.4. The Emergence of Fna C2 and Its Potential Relevance in Colorectal Cancer

With increasing taxonomic resolution, an important advance in the study of Fn heterogeneity has been the further delineation of two clades within *F. nucleatum* subsp. *animalis*, namely Fna C1 and Fna C2. Current evidence suggests that the clade more prominently enriched in the CRC tumor niche may not be the entire Fna population, but rather certain lineages that are better adapted to the tumor-associated ecological environment, among which Fna C2 has emerged as a candidate clade of particular interest [16,22]. This finding suggests that the focus of Fn–CRC research may need to move beyond describing species-level abundance changes and toward identifying lineages with potentially greater pathological relevance.

Comparative genomic analyses indicate substantial genetic divergence between Fna C2 and Fna C1. Relative to Fna C1, Fna C2 harbors more genetic features associated with adaptation to the gastrointestinal environment, including modules involved in ethanolamine utilization, 1,2-propanediol metabolism, and acid tolerance [16]. These features suggest that Fna C2 may possess greater intestinal niche adaptability, environmental resilience, and persistence, which may facilitate its selective retention and expansion in CRC-associated ecological settings [16]. By contrast, other clades may be more closely associated with the oral niche or may lack equivalent adaptive advantages in the gut [20].

From the perspective of CRC research, the identification of Fna C2 represents not only a finer taxonomic distinction, but also a more specific framework for reinterpreting the pathogenic heterogeneity of Fn. If some of the lineages most relevant in CRC are those better adapted to the tumor niche, then many conclusions previously drawn from species-level averages may primarily reflect the effects of a limited number of candidate high-risk subgroups rather than properties shared by the species as a whole. High-resolution phylogenetic analyses are consistent with this view, suggesting that different Fusobacterium lineages are not equally associated with CRC. In addition, the reported association between Fap2-positive fusobacteria and Fic gene families in CRC microbiomes further raises the possibility that specific lineages may carry more pathogenically relevant virulence gene combinations [19].

At present, however, the evidence supporting Fna C2 remains derived mainly from limited cohorts, comparative genomics, and functional inference, and therefore requires further validation through cross-cohort studies and direct functional experimentation. In parallel, the classification boundaries between Fna C1 and Fna C2, their phylogenetic interpretation, and strategies for higher-resolution detection remain open to further discussion [13,14,23]. In addition, most currently available clinical outcome data linking Fn to CRC remain at the species level, while subspecies- and especially clade-level survival analyses remain limited. Accordingly, the clinical relevance of Fna C2 should be interpreted as suggestive rather than definitively established. Nevertheless, existing studies have provided preliminary support for considering Fna C2 as a candidate high-risk lineage in CRC through multiple lines of evidence, including ecological enrichment, comparative genomics, and high-resolution lineage analysis. Representative evidence is summarized in Table 1, which is organized according to evidence domain, evidence type, biological interpretation, and major limitations, thereby highlighting that current support for Fna C2 is suggestive but not yet definitive.

## 3. Evidence Linking *Fusobacterium nucleatum* to Colorectal Cancer

Unlike many tumor-associated microbes for which the evidence remains largely limited to statistical association, the relationship between Fn and CRC is increasingly supported by a multidimensional body of evidence, including tissue enrichment, fecal detection, and clinicopathological as well as prognostic associations [7,8,24]. Current studies suggest from the perspectives of abundance, spatial organization, and potential origin that Fn is unlikely to represent a coincidental enrichment in CRC, but may instead participate in the establishment and maintenance of tumor-associated ecological niches. Accordingly, the Fn–CRC relationship should not be interpreted solely in terms of whether Fn is enriched, but rather through an integrated assessment of quantitative, spatial, and source-related evidence.

### 3.1. Quantitative Evidence: Persistent Enrichment in Tumor Tissues and Fecal Samples

One of the earliest and most consistent lines of evidence linking Fn to CRC is its persistent enrichment in both tumor tissues and fecal samples from affected patients. Previous studies have repeatedly shown that the detection rate and abundance of Fn in CRC tissues are higher than those in normal colonic mucosa or adjacent non-tumor tissues [7,24]. In fecal samples, Fn is likewise detected at relatively high frequency and appears to increase progressively along the adenoma–carcinoma sequence [25,26,27].

Importantly, enrichment of Fn in tumor tissues is not merely a matter of detectability. Higher intratumoral Fn burden has also been associated with adverse clinical outcomes, suggesting that quantitative variation in Fn may carry prognostic relevance in addition to reflecting tumor-associated microbial remodeling [8,28]. With the development of qPCR, metagenomics, and higher-resolution typing approaches, this pattern has been reproduced in larger cohorts and across independent studies, further supporting the stability and reproducibility of Fn enrichment. Quantitative evidence therefore supports not only the sustained presence of Fn in CRC, but also its potential involvement in remodeling the local tumor niche and its possible utility as a noninvasive biomarker.

### 3.2. Spatial Evidence: Colonization Within Invasive Biofilms and Organized Microbial Structures

An increase in abundance alone is insufficient to fully capture the relationship between Fn and CRC. Spatial microbiome studies indicate that in CRC, Fn is characterized not only by increased abundance, but also by altered patterns of tissue colonization [29,30]. Rather than existing as scattered luminal bacteria, Fn appears more likely to be found within invasive biofilms or organized microbial structures, where it is embedded together with other opportunistic pathogens in the tumor-associated microenvironment [30].

This spatial pattern of colonization suggests that the significance of Fn in CRC lies not only in whether it is present or abundant, but also in how it is positioned within tissue architecture. Invasive biofilms imply more sustained and intimate contact between bacteria and the host epithelium, mucus layer, stromal compartments, and immune cells, and further suggest that Fn may achieve persistent retention through adhesion, co-aggregation, and local microenvironmental remodeling. Previous studies have shown that biofilms may contribute to tumorigenesis by promoting persistent microbial colonization, altering local inflammatory states, and reshaping the tissue microenvironment [31]. Thus, in CRC, Fn may act not as an isolated bacterium, but as part of an organized pathological microbial ecosystem engaged in host–pathogen interactions. This spatial dimension also provides a more concrete pathological context for subsequent discussions of adhesion, polymicrobial interactions, and immune modulation.

### 3.3. Source-Related Evidence: The Oral–Gut–Tumor Axis and Selective Colonization

Fn is not a typical dominant member of the gut microbiota, but is more commonly regarded as an opportunistic pathogen of oral origin. On this basis, an “oral–gut–tumor axis” model has been proposed, suggesting that Fn may migrate across ecological niches under specific conditions and enter the colorectal tumor-associated niche [10,32]. Molecular typing and genomic evidence indicate that highly similar, and in some cases apparently identical, Fn strains can be detected in both oral samples and tumor tissues from patients with CRC, providing direct support for an oral origin in at least a subset of cases [32]. In addition, it has been proposed that Fn may reach the intestine through the bloodstream and subsequently colonize permissive microenvironments [33]. These observations suggest that Fn detected in CRC tissues may more likely reflect selective colonization following cross-niche migration rather than local random expansion. However, the scope of applicability and biological significance of this model still require further validation in larger cohorts and at higher taxonomic resolution (see Section 5.4).

### 3.4. Clinicopathological Associations and Limitations of the Current Evidence Framework

At the clinical level, increased Fn abundance has generally been associated with more aggressive tumor behavior, including advanced stage, lymph node metastasis, distant metastasis, increased recurrence risk, and poorer survival outcomes [8,28]. Higher-resolution studies further suggest that these adverse associations may not be contributed equally by all Fn subgroups, but may be driven more strongly by *F. nucleatum* subsp. *animalis* [34]. Multiple systematic reviews and meta-analyses likewise support an overall association between Fn and poor prognosis, suggesting that Fn may be linked not only to tumor presence, but also to disease progression and treatment outcome [35,36].

Among these clinical settings, locally advanced rectal cancer may represent one of the most informative scenarios. In this context, persistence of intratumoral Fn after preoperative therapy, particularly neoadjuvant chemoradiotherapy, has been associated with recurrence risk, suggesting that Fn-related measurements may have value not only for prognostic assessment but also for post-treatment risk assessment and follow-up stratification [28].

Nevertheless, the current evidence remains heterogeneous. Differences in the strength of reported associations may reflect variation in sample source, detection platform, anatomical site analyzed, and the spatial and strain-level resolution of the methods used. In particular, given the substantial subspecies- and clade-level heterogeneity within Fn, interpreting clinical effects solely on the basis of species-level abundance may dilute signals derived from biologically relevant high-risk subgroups and thereby obscure key host–pathogen interaction differences [13,14].

Taken together, although the association between Fn and CRC is supported by a relatively stable body of multidimensional evidence, its effect size, relevant patient populations, and clinical implications still require clarification through higher-resolution detection strategies. This in turn suggests that future studies should move beyond species-level analyses toward a more refined framework centered on functional lineages or candidate high-risk clades.

## 4. Host–Pathogen Interaction Mechanisms Underlying Tumor Promotion in CRC

The pathogenic role of Fn in CRC involves multiple virulence factors and signaling pathways and appears to exhibit strong context dependency and stage-specific characteristics [18,37,38]. These mechanisms can be broadly conceptualized as a series of interconnected processes, including adhesion and niche colonization, activation of oncogenic host signaling, amplification of inflammatory responses, establishment of an immunosuppressive microenvironment, and metabolic adaptation coupled with host metabolic reprogramming, ultimately manifesting as adverse clinical outcomes such as metastasis, recurrence, and therapy resistance.

Importantly, these processes are likely interrelated and may act in concert at different stages of disease progression. It should also be noted that most of the current mechanistic evidence has been derived from studies at the species level or using classical laboratory strains of Fn; the relative contribution of these mechanisms in specific Fn lineages remains to be clarified.

### 4.1. Adhesion, Colonization, and Tumor Niche Occupation

Selective adhesion and sustained colonization represent the initial steps in Fn-associated tumor-promoting processes. Current evidence suggests that enrichment of Fn within tumor tissues is not merely due to nonspecific bacterial attachment, but rather involves multiple defined adhesion interfaces.

Among these, several well-characterized mechanisms include FadA, Fap2, and RadD. FadA binds to E-cadherin on host epithelial cells and facilitates bacterial adhesion and invasion [39]. Fap2, a galactose-sensitive lectin, recognizes Gal–GalNAc structures that are frequently overexpressed on CRC cells, thereby enhancing tumor-specific enrichment of Fn [40]. RadD contributes not only to interbacterial adhesion and multispecies biofilm architecture [41], but may also interact with CD147 on CRC cells to promote colonization within tumor niches [42]. Collectively, these findings support the notion that Fn enrichment in tumors is an active and multi-interface process involving targeted recognition and stable colonization.

In addition to classical surface adhesins, extracellular vesicles and secreted components may also contribute to colonization and local pathogenic effects. Fn-derived outer membrane vesicles (OMVs) can fuse with host cell membranes and deliver virulence-associated components, thereby enhancing local inflammation, host interaction, and bacterial adhesion [43,44]. Outer membrane proteins such as FomA may further participate in host interaction and innate immune modulation [45]. 

However, compared with FadA, Fap2, and RadD, OMV-related mechanisms currently appear to provide a complementary rather than central explanation for colonization, and their generalizability across strains and tumor contexts remains uncertain. In addition, most studies of FadA, Fap2, and RadD have been conducted independently, and their relative contributions, potential cooperation, and hierarchical relationships remain insufficiently defined. Overall, these findings primarily support adhesion mechanisms at the species level; whether these processes are preferentially enriched in candidate high-risk lineages such as Fna C2 requires further investigation.

### 4.2. Host Signaling and Inflammatory Reprogramming

Following stable colonization, Fn may further promote tumor progression through coordinated reprogramming of host signaling pathways and inflammatory responses. Current evidence suggests that Fn can engage multiple virulence-associated interfaces to simultaneously activate oncogenic signaling pathways and pro-inflammatory responses, thereby promoting CRC cell proliferation, survival, migration, and remodeling of the tumor microenvironment [18,37,38]. From a mechanistic perspective, activation of oncogenic signaling and amplification of inflammation are not fully independent modules, but are highly interconnected through shared regulatory axes, including NF-κB activation, microRNA modulation, chemokine induction, and microenvironmental remodeling. These processes may therefore be more appropriately interpreted as a continuous module of “host signaling and inflammatory reprogramming”.

With respect to direct host signaling activation, the FadA–E-cadherin axis represents one of the best-characterized mechanisms. Binding of FadA to E-cadherin has been shown to induce ANXA1 and activate β-catenin/Wnt signaling, thereby promoting tumor cell proliferation and progression [46]. Consistently, disruption of FadA-dependent mechanisms markedly attenuates the tumor-promoting effects of Fn in experimental models, suggesting that FadA may represent a key virulence determinant in this context [39,47]. In addition to FadA, other virulence-associated interfaces also contribute to oncogenic signaling activation. For example, the RadD–CD147 axis has been reported to activate PI3K–AKT–NF-κB signaling, enhancing malignant phenotypes of CRC cells [42]. Moreover, Fn may induce miR-21 via TLR4/NF-κB signaling and activate miR-31-related programs, further promoting tumor cell proliferation and tumorigenesis [48,49].

Beyond these relatively well-established mechanisms, emerging evidence suggests that Fn may also contribute to tumor initiation-related cellular programs. For instance, Fn has been reported to localize to the base of intestinal crypts, activate LY6A^+^-associated programs, and promote transition toward stem cell-like states through the LY6A/RPS14 axis, thereby potentially contributing to CRC initiation [50]. This observation raises the possibility that the role of Fn may not be limited to promoting proliferation of established tumor cells, but may also involve earlier stages of tumor development.

In addition to direct activation of oncogenic signaling, Fn may further reshape the tumor microenvironment through sustained induction of inflammatory responses. Consistent findings primarily involve the activation of pro-inflammatory cytokines, chemokines, and NF-κB-related pathways [9,51,52]. Fn has been shown to induce the expression of IL-6, TNF-α, IL-8, CXCL1, and CCL20, thereby amplifying local inflammation and promoting recruitment of myeloid cells [9].

Beyond canonical inflammatory cytokine pathways, Fn may also enhance Th17/IL-17 responses through metabolite receptor-related mechanisms, accompanied by increased Il23p19 expression, suggesting a role for FFAR2-associated signaling in shaping a pro-inflammatory intestinal microenvironment [51]. In addition, Fn-derived pathogen-associated small molecules can further amplify inflammatory signaling. For example, the ADP-heptose–ALPK1/TIFA axis has been implicated in inducing pro-inflammatory and anti-apoptotic responses in CRC cells, while IL-8/CXCL1-related chemokine signaling has been associated with inflammatory amplification and tumor cell migration. 

In addition to soluble mediators, Fn-derived outer membrane vesicles (OMVs) may also contribute to inflammatory amplification. These vesicles can promote intestinal inflammation and function as delivery vehicles for pathogenic components, thereby enhancing local inflammatory responses, bacterial adhesion, and persistence within tumor-associated niches [43,44].

**Figure 1:**
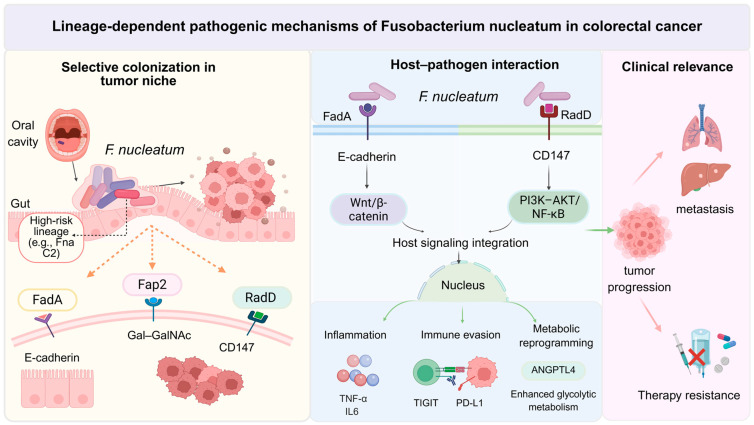
Lineage-dependent mechanisms linking *Fusobacterium nucleatum* to colorectal cancer progression. Created in BioRender. (2026) “https://BioRender.com”(accessed on 10 March 2026).

Overall, Fn-mediated activation of host signaling and inflammatory reprogramming may act as an upstream basis for subsequent immune dysregulation, metabolic adaptation, and adverse clinical outcomes, rather than as two independent pathway modules [37,53]. Accordingly, the “Host signaling integration” module in Figure 1 may be better interpreted as a conceptual summary of convergent oncogenic and inflammatory pathways rather than a distinct signaling axis. It should also be noted that most currently available mechanistic evidence has been derived from species-level Fn or representative laboratory strains [13]. Direct comparative evidence showing that different subspecies or clades preferentially activate distinct oncogenic or inflammatory host signaling programs remains limited, although clade-level ecological divergence has been reported for Fna C2 [16]. At present, lineage-specific pathway divergence should therefore be regarded as a plausible but incompletely validated hypothesis. 

### 4.3. Immune Evasion and Host Immunosuppression

Building upon a pro-inflammatory environment, Fn may further promote tumor progression by driving the transition toward an immunosuppressive tumor ecosystem. Current evidence suggests that Fn-associated immune suppression can be broadly categorized into two mechanisms: direct interaction between bacterial factors and host inhibitory receptors, and remodeling of immune checkpoint expression and immunosuppressive cellular compartments [9,54].

Among these, direct receptor interactions are relatively well supported. In addition to facilitating tumor enrichment, Fap2 can bind to TIGIT on NK cells and T cells, thereby inhibiting cytotoxic activity [55]. Similarly, the CbpF–CEACAM1 and RadD–Siglec-7 axes have been shown to suppress antitumor immune functions, expanding the range of immune regulatory interfaces utilized by Fn [56,57]. These findings suggest that Fn may simultaneously modulate both innate and adaptive immune responses through multiple molecular interactions.

Beyond direct inhibition of effector immune cells, Fn may also reinforce immunosuppressive environments by upregulating immune checkpoint molecules and reshaping myeloid compartments. For example, Fn has been reported to upregulate PD-L1/CD274 expression in CRC cells via ALPK1 signaling [58], and to promote recruitment of CX3CR1^+^PD-L1^+^ phagocytes to tumor tissues [59]. In addition, Fn may enhance macrophage infiltration and drive M2 polarization through the miR-1322/CCL20 axis [60]. Overall, Fn-associated immune suppression appears to involve coordinated remodeling of multiple immune compartments, including T cells, NK cells, and myeloid populations [61]. However, most currently available evidence for these immune-evasion effects has been derived from species-level studies or representative strains [9,54]. It therefore remains unclear whether mechanisms such as TIGIT-mediated inhibition, CEACAM1/Siglec-related suppression, or PD-L1 induction are uniformly distributed across Fn lineages or preferentially enriched in particular subspecies or clades. This represents an important knowledge gap for future lineage-resolved studies.

### 4.4. Metabolic Adaptation and Host Metabolic Reprogramming

In addition to inflammatory and immune modulation, metabolic adaptation represents an important dimension supporting both the persistence of Fn within tumor niches and its pathogenic output. Increasing evidence suggests that metabolic adaptability may contribute to differences in pathogenic potential among Fn lineages, beyond adhesion and immune modulation capacities [18]. A representative observation is that Fn may enhance its persistence in tumor niches through host metabolic reprogramming. For instance, Fn infection has been reported to upregulate ANGPTL4, thereby promoting glucose uptake and glycolysis in CRC cells, which in turn may facilitate bacterial colonization and persistence [62]. These findings suggest that Fn-associated metabolic effects may involve not only bacterial metabolism, but also modulation of host energy utilization pathways.

Additional metabolite-related mechanisms have also been proposed. Fn-derived succinic acid has been reported to suppress the cGAS–STING–IFNβ pathway and influence tumor immune responses [63]. In contrast, butyrate has been shown to inhibit Fn growth, enrichment, and adhesion, and to attenuate its tumor-promoting effects [64]. However, mechanisms such as the succinate–cGAS–STING axis remain relatively recent observations and are better regarded as candidate explanations rather than fully established models. Overall, metabolic adaptation may both support Fn persistence and amplify its pathogenic effects, but most current evidence remains at the species level, with limited direct validation at the lineage level. 

### 4.5. Metastasis, Recurrence, and Therapy Resistance as Integrated Outcomes

As colonization, signaling reprogramming, inflammation, immune suppression, and metabolic adaptation accumulate within the tumor niche, their combined effects may ultimately manifest as increased metastatic potential, recurrence risk, and therapy resistance. Thus, the pathogenic relevance of Fn may lie not only in promoting local tumor growth, but also in shaping ecological conditions that influence disease progression and treatment outcomes.

In terms of metastasis, Fn has been implicated in multiple processes, including enhanced adhesion, EMT, invasion, and remodeling of pro-metastatic microenvironments. Representative mechanisms include activation of the ALPK1–NF-κB–ICAM1 axis, which promotes tumor cell adhesion to endothelial cells and extravasation. Additional reported mechanisms include induction of MMP7 with disruption of E-cadherin function, as well as activation of KLF4/integrin α5 programs that facilitate EMT and invasive growth [65]. Additional mechanisms include regulation of KRT7-AS/KRT7 and induction of M2 macrophage polarization through the miR-1322/CCL20 axis [60,66]. Fn infection may also promote metastatic phenotypes in CRC through exosome-mediated export of miR-122-5p [67]. Recent studies have also identified Fn-Dps as a potential virulence factor that may promote EMT and metastasis through CCL2–CCL7 signaling, accompanied by increased PD-L1 expression [68]. However, this finding currently represents an early observation from a limited number of studies and requires further validation across different strains and clinical contexts.

Regarding therapy resistance, Fn has been implicated in both chemoresistance and reduced responsiveness to immunotherapy. One well-characterized mechanism involves activation of protective autophagy through the TLR4–MYD88 pathway, accompanied by downregulation of miR-18a and miR-4802 [69]. In the context of immunotherapy, Fn-derived succinate may suppress cGAS–STING signaling and reduce CD8⁺ T cell infiltration, thereby limiting responses to anti-PD-1 therapy [63]. Fn may also enhance immune evasion through upregulation of PD-L1 via ALPK1 [58]. OMVs may promote immunotherapy resistance by altering tryptophan metabolism in tumor-associated macrophages [70]. It is important to note that several of these mechanisms, particularly those involving immunometabolic regulation, remain relatively recent and should be interpreted as emerging hypotheses rather than fully established pathways.

Overall, most current evidence for Fn-mediated tumor-promoting mechanisms is derived from in vitro and animal studies, and direct causal validation in human disease contexts remains limited. Variability in strain resolution, host background, spatial context, and polymicrobial interactions further complicates interpretation of the relative contribution of individual mechanisms. Future studies integrating strain-level tracking, spatial multi-omics, functional perturbation models, and prospective clinical cohorts will be required to better define the context dependency and true effect size of Fn-associated pathogenic processes.

Taken together, the role of Fn in CRC may be better understood as a sequential and interconnected pathogenic cascade involving colonization, signaling reprogramming, inflammatory amplification, immune suppression, and metabolic adaptation. A schematic overview of this framework is presented in Figure 1.

*Fusobacterium nucleatum* (Fn) exhibits substantial intra-species heterogeneity, and accumulating evidence suggests that CRC-associated effects may be preferentially associated with specific high-risk lineages (e.g., Fna C2), rather than being uniformly distributed across the species as a whole. Under pathological conditions, Fn may translocate from the oral cavity to the colorectal tumor microenvironment, where it undergoes selective colonization and participates in structured microbial communities such as invasive biofilms.

Within the tumor niche, Fn interacts with host cells through multiple adhesins and virulence-associated factors, including FadA, Fap2, and RadD, activating key signaling pathways such as Wnt/β-catenin, NF-κB, and PI3K–AKT. These interactions contribute to inflammation, immune evasion, and metabolic reprogramming, collectively promoting tumor progression.

Importantly, these pathogenic effects are likely lineage-dependent and context-specific. Clinically, Fn enrichment has been associated with adverse outcomes, including tumor progression, metastasis, recurrence, and therapy resistance. This model highlights the need to move beyond species-level analyses toward higher-resolution, lineage-based frameworks for understanding Fn-associated tumorigenesis. 

## 5. Heterogeneous Roles of *Fusobacterium nucleatum* in CRC: Resolution, Host Context, and Ecological Conditions

Although the association between Fn and CRC has been supported by an increasing number of studies, the reported strength of this association, its immunological significance, and its clinical relevance are not fully consistent across cohorts. Taken together, such variation does not necessarily argue against a role for Fn in CRC; rather, it suggests that a species-level definition of “Fn positivity” is insufficient to capture biologically meaningful heterogeneity within the species [13,14,16].

In this context, the role of Fn in CRC may be better understood within an integrated framework shaped by risk-associated lineages, host background, polymicrobial ecology, and spatial colonization. Within such a framework, the significance of Fn is determined not only by whether it is present or abundant, but also by whether, under a given set of microbial and host conditions, it behaves more like a disease-driving factor or a conditional amplifier of pre-existing tumor-promoting processes.

### 5.1. Limited Strain-Level Resolution as a Major Source of Heterogeneity

The term “Fn-positive” does not carry a uniform biological meaning across studies. Different reports vary in their taxonomic resolution, with some remaining at the species level, others distinguishing subspecies, and only a limited number resolving specific clades or strains [13,14]. As a result, the microbial entities represented by “Fn positivity” are not necessarily comparable across cohorts, which likely contributes to inconsistency in the literature.

For CRC, the critical question is therefore not simply whether Fn is present, but which subspecies, clades, or strains are involved. Available evidence suggests that species-level detection alone is often insufficient to infer pathogenic relevance. By contrast, higher-resolution analyses that link specific lineages to colonization tendencies, ecological adaptability, and clinical associations may offer more informative biological interpretation [13,34]. In particular, *F. nucleatum* subsp. *animalis* has been associated with molecular pathological features and patient outcomes, while phylogenetic studies further suggest that certain clades may be preferentially enriched in the CRC tumor niche [16]. However, these observations should still be interpreted cautiously, as current clinical associations are more robust at the species or subspecies level than at the clade level.

Thus, differences at the clade or strain level are not merely taxonomic details, but may directly influence niche adaptation, persistence, and host interaction. Under some conditions, such lineages may be more biologically relevant than passive microbial bystanders, although their precise pathogenic contribution still requires direct lineage-resolved validation.

### 5.2. Host Immune and Molecular Context Shapes the Effects of Fn

The effects of Fn in CRC do not occur independently of host context. Current evidence suggests that Fn-high tumors are often accompanied by stronger immunosuppressive features, including impaired antimicrobial defense, weakened adaptive immunity, and reduced local antitumor activity. At the same time, host molecular events, inflammatory states, mucosal alterations, and metabolic conditions may all influence both the efficiency of Fn colonization and its downstream effects [6,34,54].

This may help explain why the reported associations between Fn and prognosis, immune status, or therapeutic response vary across studies. In particular, the immunological significance of Fn may depend on factors such as microsatellite instability (MSI) status, myeloid cell composition, local metabolic context, and the surrounding microbiota. For example, the association between Fn and immune response has been reported to differ according to tumor MSI status, suggesting that its immunomodulatory effects are strongly host-context dependent [71].

Taken together, the impact of Fn is likely determined not only by bacterial virulence traits, but also by how these traits interact with host molecular and immune conditions. Host background may therefore influence whether Fn acts more like a driver of disease progression or a conditional enhancer of ongoing tumor-promoting processes.

### 5.3. Polymicrobial Interactions and Spatial Ecology Influence Pathogenic Output

Fn-associated pathogenic effects are unlikely to occur in isolation, but rather within complex microbial communities and spatially structured ecological settings. Spatial microbiome studies have shown that Fn in CRC is preferentially associated with invasive biofilms and organized microbial structures, suggesting that its pathogenic output may depend not only on direct virulence factor activity, but also on co-colonization, spatial aggregation, and local amplification within polymicrobial communities [29,30,31].

In the oral cavity, Fn is well known as a community-bridging organism that links early and late colonizers and contributes to the maturation and spatial organization of multispecies biofilms [17]. By analogy, this feature raises the possibility that in CRC, Fn may also influence tumor-associated microbial communities by supporting co-colonization and local structural stability. However, direct evidence for such an organizer-like role in colorectal tumors remains limited.

Existing observations do provide preliminary support for polymicrobial interaction. Fn has been shown to enhance biofilm formation with other bacteria in different ecological settings, including co-aggregation with Clostridioides difficile through the RadD adhesin in intestinal mucus [72]. These findings suggest that Fn may act as a connector or amplifier within microbial communities, although current evidence does not yet support a single unified organizer mechanism in CRC.

Accordingly, the role of Fn may often be better interpreted within the context of biofilm structure and polymicrobial ecology, where it may function less as an isolated initiating agent and more as a cooperative amplifier of existing tumor-promoting processes.

### 5.4. The Oral–Gut–Tumor Axis as a Plausible but Incompletely Validated Model

The best-established natural niche of Fn remains the oral cavity. If at least a subset of CRC-associated Fn indeed originates from the mouth, then its presence in colorectal tumors should not be attributed solely to local dysbiosis, but rather to a multistep process involving cross-niche migration, environmental adaptation, and host selection [10,33].

Some studies have detected apparently identical strains in oral and tumor samples from patients with CRC, providing support for an oral origin in at least some cases [23,32]. However, this does not imply that all tumor-associated Fn follows the same migration route, nor does it prove that oral-derived strains can uniformly establish stable colonization and sustained pathogenic output after reaching the gut. More relevant questions may be which strains are best equipped to cross ecological barriers, which are most likely to be retained by the tumor microenvironment, and which host conditions facilitate these processes.

For this reason, the oral–gut–tumor axis may be better regarded as a plausible but heterogeneous explanatory model rather than a universally established pathway. Origin alone is unlikely to determine function; selective retention and persistent colonization within tumor niches may be more important in shaping whether Fn behaves as a more initiating factor or as an amplifier of pre-existing pathological processes [73].

### 5.5. Fn May Be Better Understood Along a Driver–Amplifier Continuum

Taken together, the most informative question may no longer be whether Fn is associated with CRC, but rather where it falls along a driver–amplifier continuum under different microbial, host, and ecological conditions. At present, a binary classification is likely too simplistic, as the timing, intensity, and biological mode of action of Fn may differ substantially across contexts. In this sense, these putative higher-risk subgroups are best viewed not as definitively established disease-driving populations, but as subgroups with features suggestive of greater niche adaptation, persistence, or host interaction potential in CRC-associated settings. For Fna C2, however, the currently available evidence remains strongest for ecological enrichment and inferred niche adaptation rather than for direct validation of aggressive clinical phenotypes.

Importantly, the driver–amplifier continuum proposed here is not intended as a purely conceptual hypothesis, but rather as a synthesis grounded in several recurring empirical observations. On the one hand, lineage-selective enrichment within CRC-associated niches, genomic features linked to gastrointestinal adaptation, and tumor-promoting effects observed in experimental models support the possibility that some Fn populations may behave in a more driver-like manner under specific conditions [9,16]. On the other hand, the frequent enrichment of Fn within invasive biofilms, its dependence on host background and polymicrobial context, and its capacity to amplify inflammation, immune suppression, metastasis, and therapy resistance suggest that, in many settings, it may function more as an amplifier of pre-existing tumor-promoting processes [29,30,54]. Taken together, these observations support interpreting Fn along a continuum rather than assigning it a fixed and context-independent role.

Under some circumstances, candidate high-risk lineages with stronger niche adaptability, persistence, and host interaction potential may behave more like disease-driving populations. This may be particularly relevant when they enter and adapt to tumor-associated niches early enough to influence host programs and disease progression [9,16]. However, this interpretation is still supported mainly by indirect evidence and requires longitudinal, functional, and interventional validation.

In other contexts, Fn may act more as an amplifier, not as the initiating cause of tumor-promoting imbalance, but rather as a factor that enhances inflammation, immune suppression, or polymicrobial cooperation once these processes are already established [54]. Viewing Fn through this continuum may therefore offer a more useful explanation for its heterogeneous behavior across patients, host backgrounds, and tumor ecological niches than attempting to define it as a single uniform carcinogenic entity.

**Figure 2:**
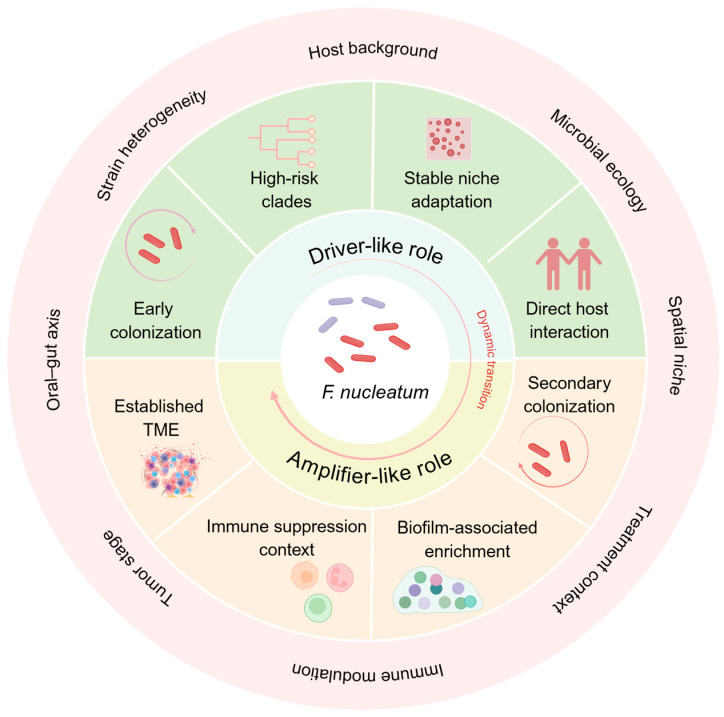
Positioning of *Fusobacterium nucleatum* along a driver–amplifier continuum in colorectal cancer. Created in BioRender. (2026) “https://BioRender.com”(accessed on 10 March 2026).

On this basis, the role of Fn in CRC may be better conceptualized as dynamic rather than fixed, shifting according to lineage characteristics, host molecular context, and microbial ecological conditions. A schematic representation of this driver–amplifier continuum is shown in Figure 2.

This schematic illustrates Fn as a dynamically positioned component along a continuum from driver-like to amplifier-like roles in CRC. Its position may be shaped by lineage characteristics, host background, microbial ecology, spatial niche, and the oral–gut axis. Driver-like states are associated with early colonization, stable niche adaptation, and stronger host interaction, whereas amplifier-like states are associated with established tumor microenvironments, immune suppression, biofilm-associated enrichment, and secondary colonization.

## 6. Clinical Translation: From Detection of Fn to Identification of Pathogenic Lineages

Fn has emerged as one of the most intensively studied microbial biomarker candidates in CRC. Repeated enrichment of Fn has been reported in tumor tissues, fecal samples, and, in some studies, oral specimens, with associations observed for tumor occurrence, poor prognosis, metastatic risk, and altered treatment response [8,26,74]. These observations suggest that Fn fulfills several basic criteria for consideration as a CRC-associated candidate biomarker.

At the same time, the translational value of Fn is unlikely to lie solely in its detectability. Rather, its potential clinical utility may span several practical scenarios, including stool-based screening support, tissue-level prognostic assessment, treatment-response stratification, post-treatment surveillance, and, in the future, lineage-informed intervention. Within this framework, moving beyond species-level detection toward identification of biologically meaningful, risk-associated lineages may be particularly important.

### 6.1. From Detectability to Composite Biomarker Utility

From an application perspective, one of the major advantages of Fn is that it is detectable using clinically accessible platforms. Current approaches include qPCR and FISH in tumor tissues, fecal real-time PCR, 16S rRNA profiling, metagenomic sequencing, and, in some cases, blood- or serum- associated assays. Existing studies suggest that combining fecal Fn with FIT, FOBT, CEA, CA19-9, or other microbial markers may improve detection performance, particularly for advanced lesions [75,76]. These features make Fn-related testing potentially relevant to practical screening-oriented workflows, especially when used in combination with established stool-based tools rather than as a stand-alone marker.

These findings suggest that the practical value of Fn is more likely to reside in composite biomarker panels than in its use as a stand-alone diagnostic marker. Fn alone has clear limitations: detection performance varies across sample types, analytical platforms, and study populations, and Fn is not specific to CRC, as it may also be detected in inflammatory bowel disease, oral disease, and other cancer-related settings. At present, Fn is therefore better viewed as an auxiliary CRC-related biomarker candidate, particularly within multi-marker frameworks, rather than as a stable and broadly applicable independent diagnostic tool. In practical terms, this supports a more realistic translational role for Fn in screening support and composite biomarker development rather than immediate use as an independent clinical diagnostic indicator.

### 6.2. From Species-Level Detection to Identification of High-Risk Lineages

Most conventional assays detect Fn at the genus or species level, essentially asking whether Fn is present. However, as discussed above, Fn exhibits substantial heterogeneity at the subspecies and clade levels, and species-level detection alone is unlikely to explain its variable pathogenic relevance across patients [13,14]. From a translational perspective, this implies that Fn-related diagnostics may need to move progressively from simple presence/absence testing toward identification of risk-associated lineages or functionally important strains.

In this context, high-resolution methods are attracting increasing attention. Compared with conventional assays, metagenomic sequencing can resolve microbial composition and functional potential at much finer taxonomic levels. At even higher resolution, CRISPR-Cas region-based strategies have enabled strain-level detection and source-tracing analyses between tumor and oral samples, thereby offering a technical basis for lineage-level interpretation [23].

In addition, large-scale integrated stool metagenomic analyses suggest that higher-resolution microbial features may show reasonable reproducibility across populations and disease stages [77]. Taken together, these developments support a shift from species-level detection toward lineage-resolved profiling of Fn. At the same time, the currently available methods differ substantially in taxonomic resolution, interpretability, and clinical readiness. These comparative features are outlined in Table 2.

### 6.3. Prognostic and Treatment-Stratification Value

Another major area of translational interest lies in the association of Fn with prognosis and treatment response. Overall, available evidence suggests that Fn is associated with poorer survival, increased recurrence risk, and several adverse clinicopathological features [8,28,36]. Fn may also influence therapeutic outcomes by reshaping immunosuppressive microenvironments, altering drug response, and weakening antitumor immunity [61]. From a practical standpoint, one of the most clinically relevant scenarios may be locally advanced rectal cancer treated with neoadjuvant chemoradiotherapy. In this setting, persistent intratumoral Fn after preoperative treatment has been associated with recurrence risk, suggesting that Fn-related measurements may eventually contribute to post-treatment risk assessment and follow-up stratification. At present, however, such use should still be regarded as exploratory rather than practice-changing, and prospective validation remains necessary before clinical implementation.

However, the prognostic and predictive value of Fn is unlikely to depend solely on overall abundance. Rather, it may be shaped jointly by lineage characteristics, host background, and treatment context. This is particularly relevant in immunotherapy-related settings, where the relationship between Fn and treatment response appears to depend on tumor molecular subtype and immune contexture. For example, the association between Fn and immune response has been reported to differ by MSI status, and the relationship between Fn and immunotherapy efficacy does not appear to be simply linear [71]. In some settings, Fn-derived succinate has been linked to resistance-associated immunometabolic effects [63], whereas other work suggests that Fn may paradoxically sensitize MSS CRC to anti-PD-1 therapy under specific conditions [78].

Accordingly, Fn-related indicators should not be interpreted as universal linear biomarkers. A more meaningful translational direction may be to integrate Fn-related features with tumor molecular classification, immune phenotype, and treatment scenario for context-specific risk stratification.

### 6.4. Targeting Fn: Interventional Strategies and Combination Approaches

The clinical relevance of Fn also extends beyond detection to its potential as a therapeutic target. Current strategies under investigation include antibiotics, drug repurposing, targeted delivery systems, microbiome modulation, and host-directed approaches [79]. Existing studies suggest that reducing Fn colonization, attenuating its tumor-promoting effects, or disrupting key host–microbe interactions may help improve the CRC microenvironment and enhance antitumor therapy [79,80,81]. From a translational perspective, these approaches suggest that Fn-related research may eventually inform more precise microbiome-directed intervention strategies rather than serving only as a descriptive biomarker framework.

Among currently available approaches, antibiotics remain the most direct intervention. Agents such as metronidazole have been reported to reduce Fn burden and partially restore treatment sensitivity in experimental models [80]. Drug repurposing strategies, including aspirin and metformin, further suggest that Fn-targeted intervention may not be limited to conventional antimicrobials [79,82]. More precise proof-of-concept approaches, such as targeted delivery platforms, may offer the advantage of reducing broad microbiota disruption while improving local therapeutic specificity [81]. However, most of these data remain preclinical, and their reproducibility and clinical applicability require further validation. At present, these strategies are better understood as emerging translational directions rather than immediately applicable clinical options.

Importantly, intervention in Fn-associated CRC is unlikely to be achieved through bacterial depletion alone. Once a stable immunosuppressive microenvironment has been established, reducing bacterial load may not be sufficient to reverse ongoing pathology. A more meaningful translational strategy may therefore involve combining microbial modulation with metabolic correction and immune reprogramming in order to indirectly constrain Fn-associated pathogenic effects [79,80,83,84].

For this reason, empirical intervention for all Fn-positive patients is unlikely to be appropriate. A more rational approach would be to identify potentially high-risk lineages and then interpret them in conjunction with tumor molecular subtype, immune status, and treatment context. Overall, Fn-related intervention appears to be moving from empirical bacterial depletion toward a more integrated strategy centered on high-risk lineage identification, blockade of pathogenic interfaces, and remodeling of the host microenvironment. Its clinical implementation, however, will depend on improved stratification systems, practical delivery platforms, and prospective validation [79].

## 7. Conclusions and Future Perspectives

### 7.1. Conclusions

Overall, the role of Fn in CRC should no longer be reduced to a simple phenomenon of species-level enrichment. Current evidence more strongly supports interpreting Fn as a tumor-associated opportunistic pathogen with substantial internal heterogeneity, whose biological significance is jointly shaped by host background and ecological context. In other words, the pathogenic relevance of Fn in CRC is unlikely to represent a uniform effect that operates consistently across all patients and all tumor niches, but instead appears to depend on the combined influence of lineage characteristics, host molecular context, polymicrobial interactions, and spatial ecological organization.

Accordingly, future investigation of Fn should move beyond the species-level question of whether it is enriched and instead focus on which candidate high-risk lineages are selectively retained in tumor niches, which host conditions favor amplification of their pathogenic effects, and which host–pathogen interaction interfaces are most relevant to biological and clinical outcomes. In this regard, Fn provides not only a representative model for understanding tumor-associated opportunistic pathogens, but also a conceptual framework for moving CRC microbiome research from coarse association analysis toward higher-resolution mechanistic dissection and stratified intervention.

Particularly important is the emerging emphasis on higher-resolution lineage identification, definition of key virulence-associated interfaces, and interpretation within host- and niche-dependent frameworks. Together, these advances provide a more operational basis for Fn-related biomarker development, risk stratification, and precision intervention. Ultimately, only by integrating high-resolution strain typing, mechanistic validation, and clinical stratification will it be possible to define more accurately the pathogenic significance of Fn and its practical relevance in CRC prevention, monitoring, and treatment.

### 7.2. Future Perspectives

Although the association between Fn and CRC is now supported by a relatively stable body of evidence, the major challenge in the field is no longer to accumulate more “Fn-related” observations, but rather to resolve key questions related to causal interpretation, mechanistic heterogeneity, and clinical stratification. Several priorities appear particularly important.

First, it remains unclear whether the major pathogenic effects attributed to Fn are actually driven by specific high-risk clades or strains rather than by an average species-level signal. Comparative genomics and high-resolution phylogenetic studies suggest that the lineages enriched in CRC may not be arbitrary Fn populations, but rather subsets with greater adaptability to the gastrointestinal tract and tumor niche, among which Fna C2 currently represents a prominent candidate for further testing. However, this inference still relies mainly on ecological enrichment, comparative genomics, and limited functional observations, while direct clinical and mechanistic validation at the clade level remains limited. Future work should therefore combine cross-cohort replication, lineage-resolved detection strategies, and isogenic or closely related strain-comparison models to determine which lineages most plausibly represent candidate high-risk populations in CRC.

Second, the pathogenic effects of Fn should not be viewed as fixed intrinsic bacterial properties independent of context. Instead, they need to be interpreted in conjunction with host molecular background, immune status, polymicrobial communities, and spatial ecological conditions. A more informative question for future studies is therefore not merely whether Fn is enriched, but under what host conditions, through which lineage, by means of which interaction interface, and at which spatial location Fn can sustain biologically meaningful pathogenic output. Only through genuine integration of high-resolution strain profiling, spatial localization, mechanistic validation, and clinical stratification can Fn move from a correlation-based biomarker toward a more explanatory and clinically useful pathogenic stratifier.

Third, the role of Fn in the real tumor niche is unlikely to be fully captured by single-species infection models. Polymicrobial interactions, invasive biofilms, and spatial tissue organization may jointly determine the stability of colonization, the degree of inflammatory amplification, and the intensity of immune suppression. Future studies should therefore more extensively incorporate spatial omics, lineage tracking, polymicrobial co-culture systems, and in situ models in order to better approximate the biological complexity of the native tumor ecosystem.

Fourth, although the oral–gut–tumor axis has become an important framework for understanding the origin and migration of Fn, key issues related to source attribution, migration route, ecological filtering, and host permissiveness remain unresolved. Longitudinal sampling, phylogenetic tracking, and spatial evidence will be needed to determine which strains are able to cross ecological barriers, persist within tumor-associated environments, and exert sustained biological effects.

Fifth, the clinical translation of Fn-related research still needs to progress from detection to stratification. A more meaningful future direction is unlikely to be empirical bacterial depletion in all Fn-positive patients, but rather the development of more precise strategies that integrate high-risk lineage identification, blockade of key pathogenic interfaces, and remodeling of the host microenvironment. Only within a higher-resolution translational framework will it be possible to define more accurately the pathogenic significance of Fn and its practical utility in CRC prevention, monitoring, and treatment.

## Figures and Tables

**Table 1:** Representative lines of evidence supporting Fna C2 as a candidate high-risk clade in colorectal cancer.

Evidence Domain	Evidence Type	Key Observation	Interpretation	Main Limitation	Strength of Support	Key References
Ecological enrichment	Clade-level ecological association	The CRC-associated tumor niche appears to be enriched for a distinct Fna-related clade, rather than for the entire Fna population as a whole	Supports the possibility that CRC-associated colonization is clade-selective and that Fna C2 represents a candidate high-risk lineage	Current support is still derived mainly from limited cohorts and clade-level stratification analyses; cross-cohort consistency remains to be established.	Moderate	[16,19]
Genomic divergence	Comparative genomics	Fna C2 differs from Fna C1 at the genomic level and carries more traits linked to gastrointestinal adaptation	Suggests that Fna C2 may have a distinct ecological adaptation basis compared with other clades	Direct causal links between these genomic features and pathogenic phenotypes remain insufficiently validated.	Moderate	[13,16]
Metabolic adaptation	Inferred functional potential	Ethanolamine utilization, 1,2-propanediol utilization, and acid-resistance modules are more prominent in Fna C2	Suggests a potential advantage for persistence and selective retention in the gastrointestinal tract and tumor microenvironment.	Current support is based largely on inferred metabolic potential rather than direct in vivo functional validation.	Moderate	[16,21]
Virulence-related response	Limited in vitro functional observation	Specific metabolic conditions can induce upregulation of virulence-associated genes in Fna C2	Raises the possibility that microenvironmental cues may further amplify pathogenic phenotypes in this clade.	Evidence remains largely inferential and based on in vitro observations; in vivo relevance remains unclear.	Preliminary	[16]
Clinical association	Indirect clinical correlation	The animalis subspecies has been associated with molecular pathological features and adverse outcomes in CRC.	Provides indirect clinical support that some animalis-related subgroups may carry greater risk relevance	This is subspecies-level rather than clade-level evidence and cannot be regarded as independent clinical validation of Fna C2.	Indirect	[13]

Note: This table summarizes representative rather than definitive lines of evidence supporting Fna C2 as a candidate high-risk clade in CRC. Current support is derived mainly from ecological enrichment, comparative genomics, inferred metabolic potential, and limited functional observations. The assigned level of support reflects the biological specificity and directness of the available evidence rather than the prestige of the publication source. Importantly, most currently available clinical outcome data linking Fn to CRC remain at the species level, and direct clinical as well as mechanistic validation at the clade level remains limited.

**Table 2:** Detection and stratification strategies for *Fusobacterium nucleatum* in colorectal cancer: from species-level detection to higher-resolution risk stratification.

Strategy Type	SAMPLE TYPE	Main Method	Target Readout	Main Strengths	Main Limitations	Resolution Level	Clinical Readiness	Typical Applications
Species-level detection	Stool	qPCR	Fn abundance	Noninvasive, fast, scalable	Cannot distinguish high-risk clades or functional heterogeneity	Low	High	Screening
Species-level detection	Tissue	qPCR	Intratumoral Fn burden	Sensitive, direct	Invasive; provides no spatial context and no clade-level information	Low	Moderate	Tissue validation
Spatial localization	Tissue	FISH	Spatial distribution	Preserves spatial context and supports biofilm-related interpretation	Limited functional and clade-level resolution	Low–Intermediate	Moderate	Biofilm studies
Serologic detection (pan-Fn)	Serum	ELISA	Anti-Fn IgA	Noninvasive, convenient for population-level studies	Reflects exposure rather than direct tumor-associated colonization; limited specificity	Low	Low–Moderate	Population studies
Serologic detection (FadA)	Serum	ELISA	Anti-FadA IgA	More mechanism-linked than pan-Fn serology	Still indirect; does not confirm tissue colonization or clade identity	Low–Intermediate	Low–Moderate	Auxiliary biomarker
Combined screening	Stool	FIT + Fn qPCR	Combined signal	Improves detection performance when added to established stool-based tools	Performance depends on cohort composition, cutoffs, and model design	Low	Moderate–High	Screening
Metagenomics	Stool or tissue	Sequencing	Community + function	Broad ecological and functional information; higher interpretability than single-marker assays	Expensive, computationally complex, and not yet scalable for routine use	High	Low	Research
Subspecies/clade typing	Stool or tissue	High-resolution typing	Fna C2 and related lineages	Better links detection to pathogenic heterogeneity and mechanistic interpretation	Lack of standardized assays, thresholds, and prospective validation	High	Low	Stratification research
Multi-marker models	Stool	Combined qPCR	Fn + others	More robust than single-marker strategies	Model complexity and cross-cohort reproducibility remain concerns	Intermediate–High	Low–Moderate	Risk models
Functional-locus detection	Stool or tissue	Targeted PCR	FadA, Fap2	Provides mechanism-linked information beyond total abundance	Detecting virulence-related loci is not equivalent to confirming clade identity or in vivo phenotype	Intermediate	Low	Mechanistic support
Integrated framework	Multi-source	Multi-omics integration	Clade, function, host background, and microenvironment	Highest biological interpretability and precision-stratification potential	Not yet standardized and currently confined to research settings	Very high	Exploratory	Future direction

Note: This table summarizes commonly used approaches for detecting and stratifying Fn in CRC across different sample types and analytical resolutions. The listed methods differ substantially in what they capture, ranging from simple species-level abundance to spatial distribution, serologic exposure, community composition, and higher-resolution lineage-related features. “Resolution level” refers to the extent to which a method can distinguish biologically meaningful heterogeneity beyond species-level detection, whereas “clinical readiness” reflects current feasibility for broader translational application rather than definitive clinical validity. In general, higher-resolution approaches may offer greater biological interpretability, but they currently remain less standardized and less readily scalable for routine clinical use.

## Data Availability

No datasets were generated or analyzed during the current study.

## Abbreviations

The following abbreviations are used in this manuscript:

Abbreviation	Definition
CRC	colorectal cancer
Fn	*Fusobacterium nucleatum*
Fna	*Fusobacterium nucleatum* subsp. *animalis*
Fna C1/C2	*Fusobacterium nucleatum* subsp. *animalis* clade 1/clade 2
IBD	inflammatory bowel disease
FadA	*Fusobacterium* adhesin A
RadD	arginine-inhibitable adhesin
OMVs	outer membrane vesicles
TLR4	Toll-like receptor 4
NF-κB	nuclear factor kappa B
PI3K	phosphoinositide 3-kinase
AKT	protein kinase B
Wnt	Wingless/Integrated signaling pathway
miRNA	microRNA
miR-21	microRNA-21
miR-31	microRNA-31
IL	interleukin
TNF-α	tumor necrosis factor alpha
CXCL	C-X-C motif chemokine ligand
CCL	C-C motif chemokine ligand
FFAR2	free fatty acid receptor 2
ALPK1	alpha-kinase 1
TIFA	TRAF-interacting protein with forkhead-associated domain
MSI	microsatellite instability
FISH	fluorescence in situ hybridization
qPCR	quantitative polymerase chain reaction
FIT	fecal immunochemical test
FOBT	fecal occult blood test
CEA	carcinoembryonic antigen
CA19-9	carbohydrate antigen 19-9
